# Design of UWB Monopole Antenna with Dual Notched Bands Using One Modified Electromagnetic-Bandgap Structure

**DOI:** 10.1155/2013/917965

**Published:** 2013-09-19

**Authors:** Hao Liu, Ziqiang Xu

**Affiliations:** ^1^Research Institute of Electronic Science and Technology, University of Electronic Science and Technology of China, Sichuan 611731, China; ^2^State Key Laboratory of Electronic Thin Films and Integrated Devices, University of Electronic Science and Technology of China, Sichuan 611731, China

## Abstract

A modified electromagnetic-bandgap (M-EBG) structure and its application to planar monopole ultra-wideband (UWB) antenna are presented. The proposed M-EBG which comprises two strip patch and an edge-located via can perform dual notched bands. By properly designing and placing strip patch near the feedline, the proposed M-EBG not only possesses a simple structure and compact size but also exhibits good band rejection. Moreover, it is easy to tune the dual notched bands by altering the dimensions of the M-EBG. A demonstration antenna with dual band-notched characteristics is designed and fabricated to validate the proposed method. The results show that the proposed antenna can satisfy the requirements of VSWR < 2 over UWB 3.1–10.6 GHz, except for the rejected bands of the world interoperability for microwave access (WiMAX) and the wireless local area network (WLAN) at 3.5 GHz and 5.5 GHz, respectively.

## 1. Introduction

UWB technology has received great attentions from both the academic and the industrial sectors since the US Federal Communications Commission (FCC) authorized the unlicensed use of UWB from 3.1 to 10.6 GHz for commercial communication purposes. As an important component of UWB system, UWB antenna has attracted increasing attentions. A number of UWB antennas with different geometries have been experimentally characterized [1–4]. Among them, planar monopole antennas are considered as good candidates for UWB applications due to their attractive merits, such as large impedance bandwidth, easy fabrication, and omnidirectional radiation pattern [5].

Many kinds of UWB planar antennas are designed to satisfy the requirements of UWB operation. However, in practical applications, some other existing narrowband services that already occupy frequencies in the UWB may cause potential interference, for instance, WiMAX for some European and Asian countries (3.3–3.6 GHz) and WLAN for IEEE802.11a in the USA (5.15–5.35 GHz and 5.725–5.825 GHz). Furthermore, C-band system (3.7–4.2 GHz) and X-band satellite communication services from 7.25 to 8.395 GHz (downlink: 7.25–7.745 GHz, uplink: 7.9–8.395 GHz) also operate in the UWB [6, 7]. So, it is necessary to design UWB antennas with multiband filtering functionality to protect the UWB system from any interference among them. Various UWB antennas with band-notched characteristics to alleviate potential electromagnetic interference have been reported recently [8–18]. Usually, there are two main methods used in the design of notched band. The conventional method focuses on cutting various slots in the patch/ground plane, such as L-shaped slot [8], open-ended slot [9], semicircular slots [10], U-shaped slot [11], square-shaped slot [11], *Ω*-shaped slot [13], *π*-shaped slot [14], fractal slot [15], and complementary edge-coupled SRR-shaped slot [16]. Another effective method is loading diverse parasitic elements on the antennas, such as L-shaped or ring-shaped branches near ground [17, 18], various strips near patch [19], and split-ring resonators (SRRs) or stepped-impedance resonators (SIRs) near feedline [20, 21]. Generally, one slot/parasitic element can only generate one notched band and fails to meet the requirements of avoiding multiple interferences caused by the coexisting systems. In order to design dual/multinotched UWB antenna, multielements are commonly needed. Although these methods can design UWB antennas with high performances, they still unavoidably exhibit some inherent shortcomings in practical applications, such as bringing occupation of too large space on the antenna as well as strong coupling between band notched structures. Therefore, implementing those approaches accordingly caused complicated design. Under the circumstances, the mushroom-style EBG structures are introduced to be another solution to design UWB antennas with multiple suitable band notches and compact sizes [22].

Recently, EBG structures have been implemented in different applications because they have characteristics of surface wave reduction, eliminating spurious response [23, 24]. Various types of these structures have been used in the design of UWB antennas with band-notched characteristics [25–30]. In [28], EBG structure is applied to a UWB wide-slot antenna to obtain narrow notched band. In [29], both the conventional mushroom-type EBG and edge-located via mushroom-type EBG have been studied to design notched band. Nevertheless, all of the above researches proposed that one EBG cell can only obtain a notched band; dual notched bands can only be achieved by two EBG structures with different sizes, which will occupy more surfaces of antennas. In [30], the EBG structure which comprises a slotted patch and an edge-located via is proposed to obtain dual notched bands. However, a patch with two L-shaped slots still makes it complicated to tune the two notched bands. Similarly, the EBG structure with quadrate patch also occupies too much space.

In this paper, one modified electromagnetic-bandgap (M-EBG) structure is used to generate dual notched bands in a common UWB antenna. The proposed M-EBG is more compact and simple. Moreover, it is convenient to control the notched bands by changing the dimensions of the M-EBG flexibly. An experimental UWB antenna with the M-EBG structure placed near the feedline is fabricated and measured for WiMAX and WLAN applications. Measured results are provided to show good performance and to be in agreement with the simulated ones, where two band notches with little effect on the radiation patterns are obtained.

## 2. Proposed M-EBG

**Figure 1:**
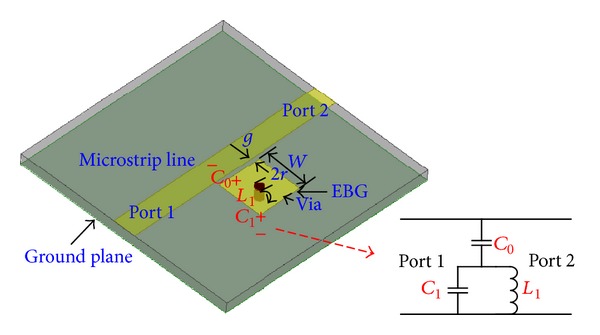
3D overviews and equivalent-circuit model of EBG.

The EBG has been studied before in [22, 23], which verified that it has a bandstop property.  Figure 1 shows the 3D overviews and equivalent-circuit model of EBG. The EBG structure is formed by a via-loaded metal patch to the ground, and it can be characterized by an equivalent LC resonator, equivalent-circuit model of EBG is also shown in Figure 1. According to the study in [22], the resonant frequency of the EBG cell which is also the center frequency of the notched band can be defined by the following equation:

(1) $$\begin{array}{c} f_{r}=\frac{1}{2\pi \sqrt{L_{1}\left(C_{0}+C_{1}\right)}}, \end{array}$$

where capacitance *C*_0_ denotes the coupling between the EBG structure and the feedline, while the capacitance *C*_1_ represents the voltage gradients between the patch and the ground plane. The inductance *L*_1_ is due to the current flowing through the shorting pin, respectively.

**Figure 2:**
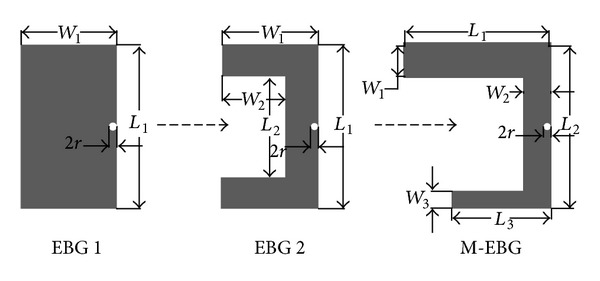
Evolution of the M-EBG.

As a result, the notched band can be achieved at the desired frequency by appropriately adjusting dimensions of EBG. In [29], an edge-located via mushroom-type EBG structure has been studied, as shown in Figure 2, and denoted as EBG 1. The edge-located via mushroom-type EBG has advantage of compactness and better frequency rejection over the conventional mushroom-type EBG when it is applied to the design of UWB notched-band antenna. So, when further design is desired, the edge-located via mushroom-type EBG is preferred. In order to achieve dual notched bands without increasing the number of EBG structures, an M-EBG based on the edge-located via mushroom-type EBG is proposed. Figure 2 shows the evolution of the M-EBG. These EBG structures are denoted as EBG 1, EBG 2, and M-EBG, respectively. As depicted in Figure 2, the EBG cell is coplanar with the microstrip line with a gap of *g*. The radius of the via is *r*. The parameters for EBG 1, EBG 2, and, proposed M-EBG are summarized in Table 1.

**Figure 3:**
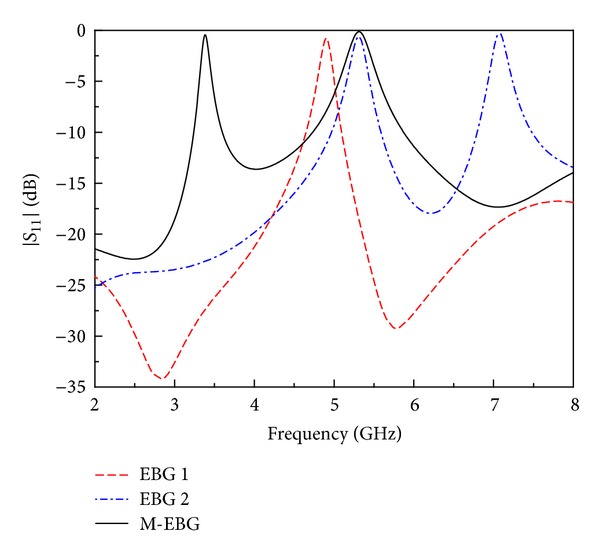
Comparison of the simulated return loss among EBG 1, EBG 2, and the proposed M-EBG.

Figure 3 shows the simulated return loss of EBG 1, EBG 2, and M-EBG. It can be seen that when cutting slot on EBG 1 patch, it becomes EBG 2. It is observed that EBG 2 can obtain two stopbands, but the bandwidths of two stopbands are the same. It is clearly seen that the M-EBG performs two stopbands of which the first one has a narrower bandwidth than the second one and retains good rejection effect. In order to achieve the notched band at the desired frequency with the desired bandwidth, we can change the length and width of the strip.

## 3. UWB Antenna with M-EBG

**Figure 4:**
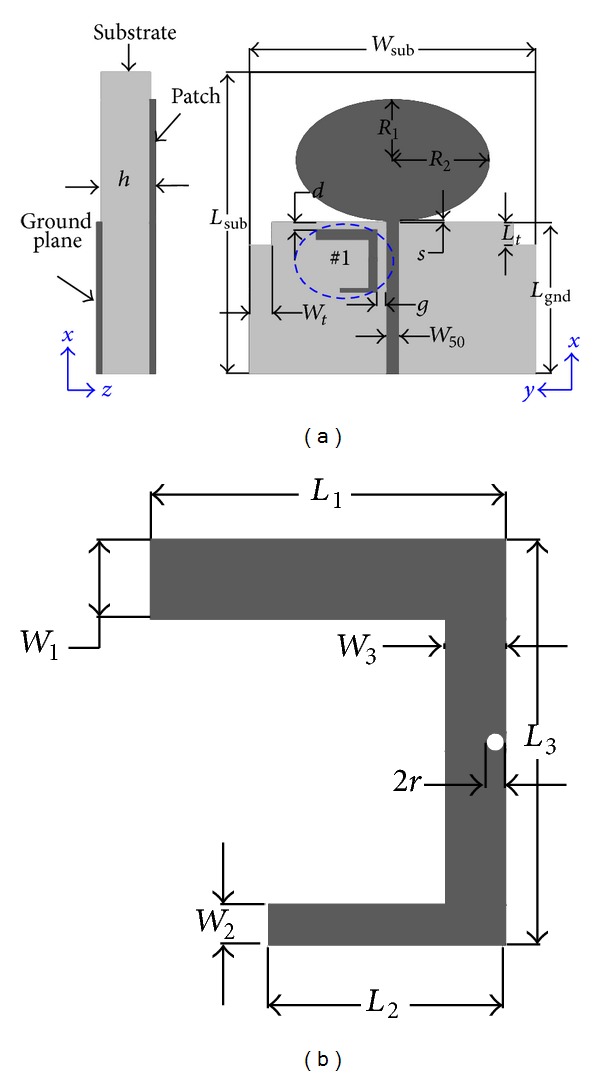
(a) Top view and side view of the proposed antenna. (b) #1 (M-EBG).

The geometrical configurations of the proposed antenna are shown in Figure 4(a). The antenna achieves notched bands property by introduction of the M-EBG structure, which is shown in Figure 4(b). The proposed antenna is fabricated on an FR4 substrate with relative dielectric constant of 4.4, loss tangent of 0.02, and thickness of 1.0 mm; the overall size of the antenna is 38 × 40 mm^2^. As shown in Figure 4(a), the elliptical radiator patch is fed by a 50 *Ω* microstrip line on one side of the substrate. On the other side of the substrate, the rectangular ground plane with two slots to enhance the impedance bandwidth; *L*_gnd_ is length of the ground plane which covers the section of the microstrip feedline. *S* is the width of the gap between the ground plane and elliptical patch. The distance between the upper edge of the ground plane and the EBG is *d*. The optimized dimensions of the proposed antenna to achieve a UWB (VSWR ≤ 2) are listed in as follows: *W*_sub_ = 38 mm, *L*_sub_ = 40 mm, *L*_gnd_ = 20 mm, *R*_1_ = 8 mm, *R*_2_ = 12.8 mm, *S* = 0.2 mm, *d* = 0 mm, *w*_50_ = 1.86 mm, *L*_*t*_ = 3.0 mm, *W*_*t*_ = 3.0 mm, *h* = 1.0 mm, *r* = 0.2 mm, *W*_1_ = 1.2 mm, *L*_1_ = 8.0 mm, *W*_2_ = 0.4 mm, *L*_2_ = 4.8 mm, *W*_3_ = 1.0 mm, and *L*_3_ = 8.2 mm.

**Figure 5:**
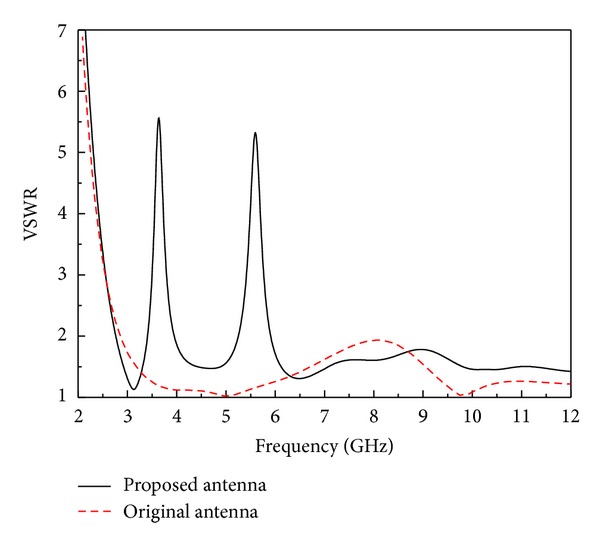
Simulated VSWRs of the proposed antenna and the original antenna.

Figure 5 shows the simulated VSWRs of the proposed UWB antenna and the original antenna. The impedance bandwidth (VSWR ≤ 2) of the original antenna is 3–12 GHz. Compared with the original antenna, it can be seen that the proposed antenna had an impedance bandwidth (VSWR ≤ 2) of 2.8–12 GHz, with two effective notched bands of WiMAX in the frequency band of 3.6 GHz (3.36–3.85 GHz, VSWR > 2) and WLAN around 5.5 GHz (5.2–5.9 GHz, VSWR > 2).

## 4. Parametric Analyses

**Figure 6:**
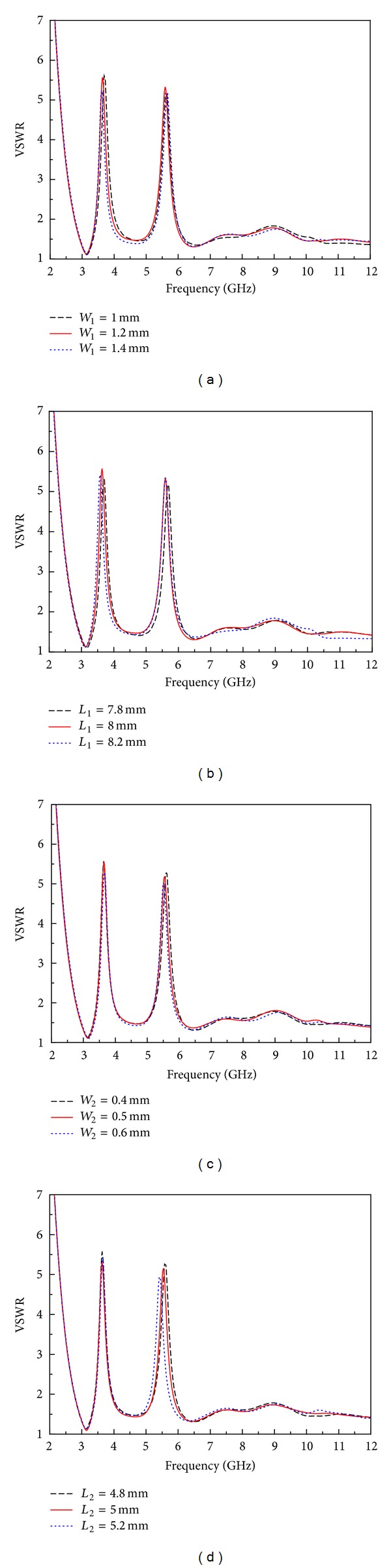
Effects of the parameters of M-EBG on the notched bands: (a) *W*_1_, (b) *L*_1_, (c) *W*_2_, and (d) *L*_2_.

In order to find out how different dimensions of M-EBG affected the center frequency and impedance bandwidth of the notched band, a parametric study is presented by using computer simulation. Notice that, when one parameter is changed, the others are fixed. As demonstrated in reference [29], the width and the length of EBG patch are directly related to the notch frequency, while the parameters *g* and *d* are used to tune the width of the notched band, respectively. For simplicity, these results are not shown in this paper. Four parameters (*W*_1_, *L*_1_, *W*_2_, and *L*_2_) of the proposed antenna are studied as depicted in Figure 6.

**Figure 7:**
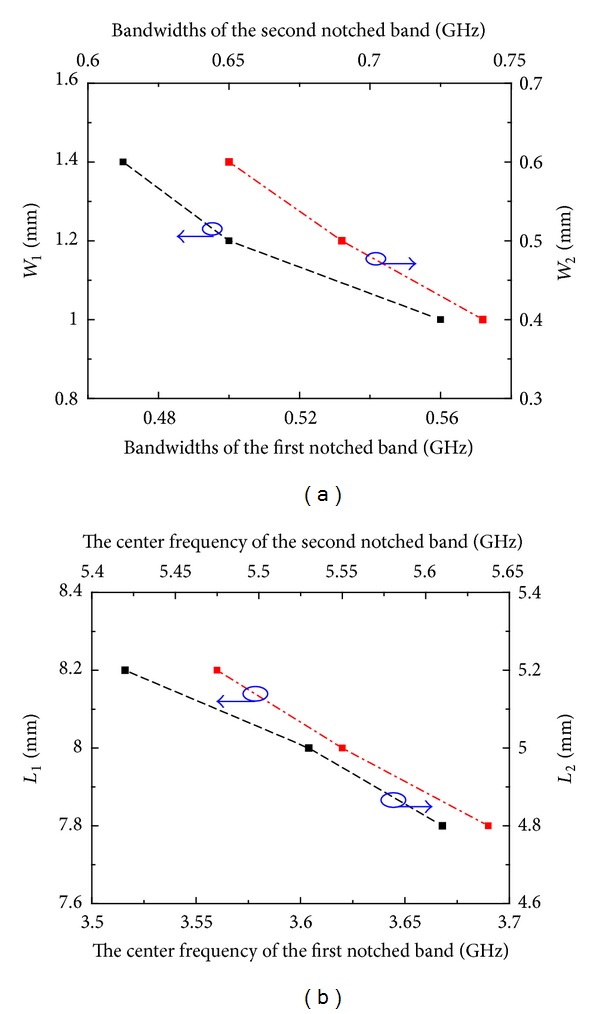
Detailed effects of the parameters of M-EBG on the notched bands: (a) *W*_1_ and *W*_2_ (b) *L*_1_ and *L*_2_.

Simulated VSWR curve with different values of *W*_1_ and *L*_1_ are shown in Figures 6(a) and 6(b). It can be indicated from Figure 6(a) that the lower notched bandwidth was inversely proportional to the width of *W*_1_, while the result in Figure 6(b) illustrates that the lower notched frequency was effectively tuned along with the length of *L*_1_. Thus for lower notch, the dimensions *W*_1_ and *L*_1_ could be used to set the notched frequency and bandwidth which little affect the rest of the UWB. In Figures 6(c) and 6(d), VSWR curve with different values of  *W*_2_ and *L*_2_ are illustrated. As depicted in the figure, with the change of parameters *W*_2_/*L*_2_, the bandwidth and the center frequency of the second notched band can be tuned effectively, respectively, which have no effect on the first notched band. Detailed effects of the parameters of M-EBG on the notched bands are shown in Figure 7. It is clearly seen that the bandwidth of the notched band can be easily tuned by *W*_1_/*W*_2_ (as shown in Figure 7(a)).  Figure 7(b) also shows that the center frequency of notched band can be effectively tuned along with the length of *L*_1_/*L*_2_. So for the presented antenna, the dimensions *W*_1_, *L*_1_, *W*_2_, and *L*_2_ could be used to set the frequency and bandwidth of notched bands. The results of the parametric discussion demonstrated that the notched frequency and bandwidth could be effectively set using the dimensions of M-EBG. This property provides antenna designers with an easy and convenient method to design compact antenna with dual band-notched characteristics at the desired frequency with the desired bandwidth.

## 5. Results and Discussion

**Figure 8:**
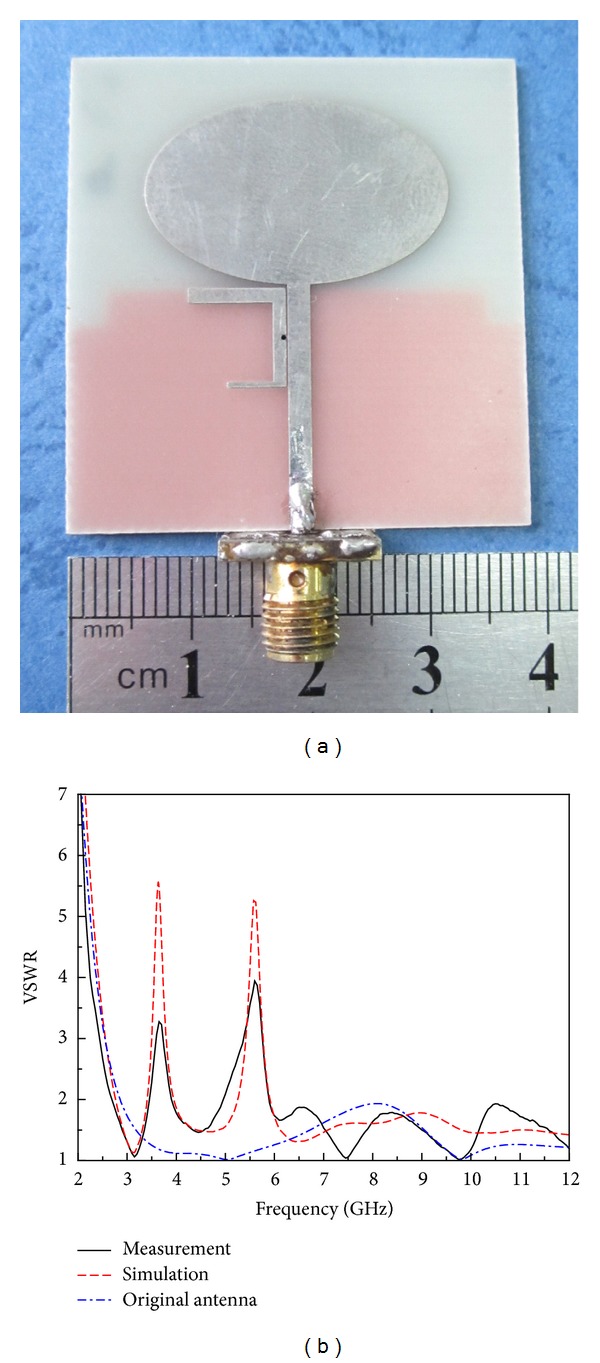
(a) Photograph of prototyped antenna. (b) Simulated and measured VSWR of the proposed antenna.

To verify the simulated result shown in Figure 5, the presented antenna has been fabricated on an *h* = 1.0 mm height FR4 substrate with relative dielectric of 4.4 and loss tangent of 0.02, as shown in Figure 8(a), using the dimensions listed previously.  Figure 8(b) displays the simulated and measured VSWR of the proposed UWB antenna with two notched bands, where the original UWB antenna without notched band is also given as a reference. It is clearly seen from the figure that a good agreement was reached between the simulated and measured results. The measured results show two effective notched bands of WiMAX around 3.6 GHz and WLAN in the frequency of 5.5 GHz (4.95–5.9 GHz, VSWR > 2), a few deviations of second notch between simulation and measurement. This is mainly caused by the fabrication tolerances and difference between the actual and nominal values of the relative dielectric constant. In addition, it should be mentioned that dielectric loss of the substrate and the influence of the transition between the joining of SMA connector and microstrip will also result in the discrepancy in the simulated and measured VSWR.

**Figure 9:**
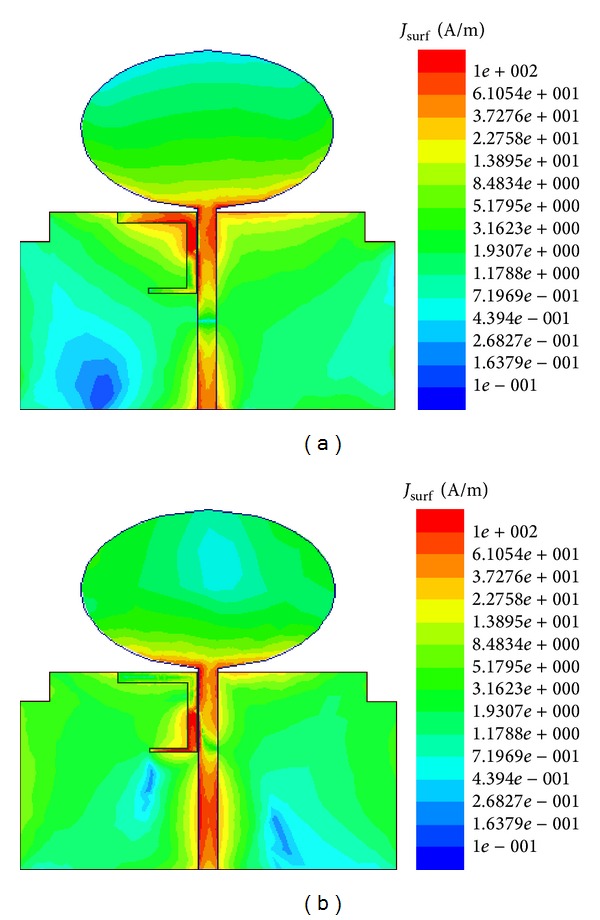
Surface current distribution at (a) 3.6 GHz and (b) 5.5 GHz.

Figure 9 displays the simulated surface current distributions at two center frequencies of notched band at 3.6 GHz and 5.5 GHz. It is indicated from the figure that the M-EBG with two short strips is corresponding to two notched bands. When the antenna is working at the center of the first notch around 3.6 GHz, most current distributions focus on the upper L-type short strip (as shown in Figure 9(a)), which means that it resonates near 3.6 GHz; therefore, the energy cannot be radiated effectively and leads to a notched band around 3.6 GHz. Similarly, the lower L-type short strip operates as a second notch at the center frequency band around 5.5 GHz as shown in Figure 9(b).

**Figure 10:**
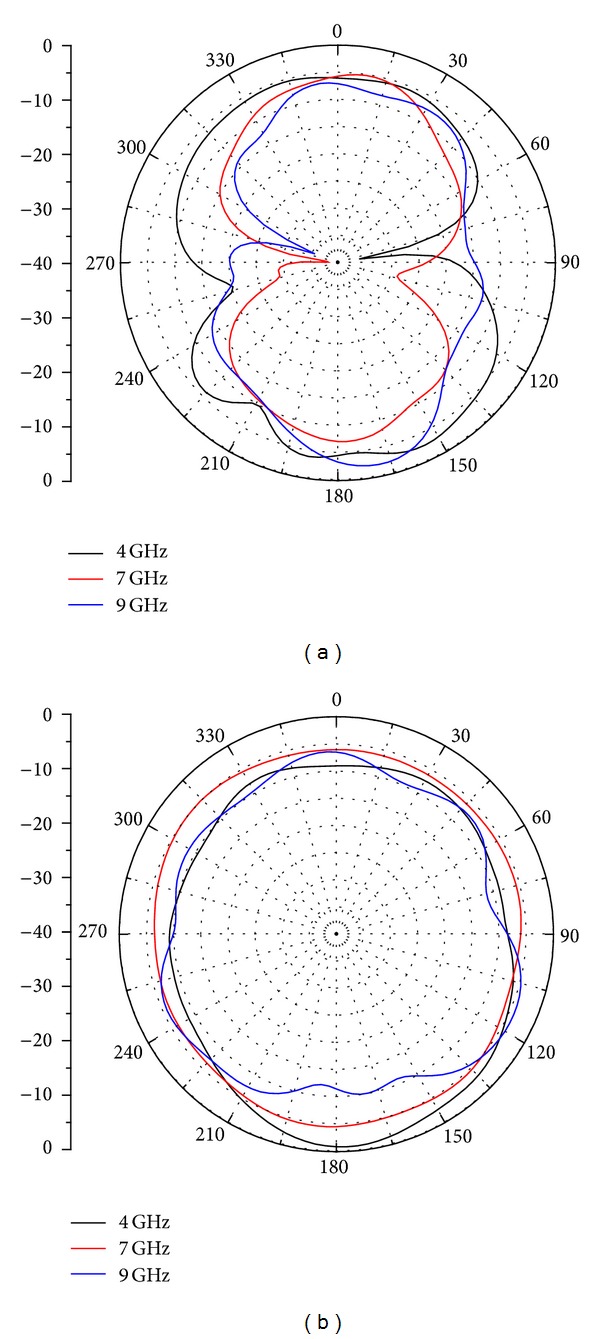
Measured radiation patterns at (a) E-plane and (b) H-plane.

**Figure 11:**
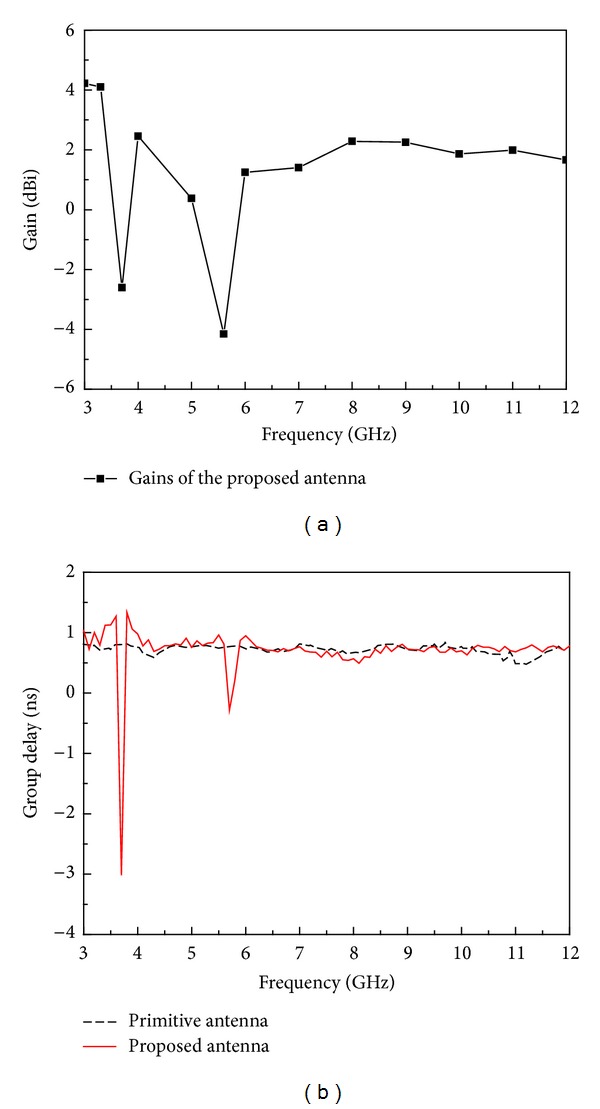
(a) Measured gain and (b) group delay of the proposed antenna.

The measured radiation patterns of the presented antenna are also given in this paper. The normalized radiation patterns of the proposed antenna at 4, 6, and 9 GHz in *x*-*z* plane and *y*-*z* plane are displayed in Figure 10. It is clearly seen from the figure that the proposed antenna exhibits a nearly omnidirectional radiation pattern in *y*-*z* plane (H-plane) similar to monopole antenna in *x*-*z* plane (E-plane). The maximum gain of the dual band-notched antenna is measured and exhibited in Figure 11(a). It is noted that the proposed antenna exhibits flat gain over the operating band, except a sharp decrease in each notched band, which is due to the frequency band-notched characteristics. Finally, the group delay of proposed antenna has also been measured, as shown in Figure 11(b). In the experiment, the distance between the two antennas was 30 cm, which obtains the far-field condition of the antenna. The group delay is about 1 ns across the frequency band except in the notched bands, due to the band-notched function. For the rest of the frequency band, the group delay characteristic is relatively flat, indicating that the antennas have good linear transmission performances.

## 6. Conclusion

A new approach to obtain dual notched bands using a single M-EBG structure has been proposed. The M-EBG consists of two L-shaped strips with different dimensions. The design method is performed simply by placing the M-EBG structure closed to the microstrip feedline; two required notched bands at WLAN and WiMAX are achieved. The notched bands are also tunable by adjusting the dimensions of the M-EBG structure. The analysis results show that the proposed antenna guarantees a bandwidth from 3.1 to 10.6 GHz and can obtain two desired dual notched bands. Furthermore, it keeps omnidirectional radiation performance successfully. The performance of the proposed antenna demonstrates that it is suitable for UWB applications.

## Figures and Tables

**Table 1:** Dimension parameters of EBG 1, EBG 2, and M-EBG (unit: mm).

Parameter	*W*_1_	*L*_1_	*g*	*r*	*W*_2_	*L*_2_	*W*_3_	*L*_3_
Type								
EBG 1	5	5	0.2	0.2	—	—	—	—
EBG 2	5	5	0.2	0.2	4.0	3.0	—	—
M-EBG	1.2	8	0.2	0.2	1.0	8.2	0.4	4.8
